# Size Variation in Small-Bodied Humans from Palau, Micronesia

**DOI:** 10.1371/journal.pone.0003939

**Published:** 2008-12-17

**Authors:** Andrew Gallagher

**Affiliations:** School of Anatomical Sciences, University of the Witwatersrand, Parktown, Johannesburg, South Africa; University of Utah, United States of America

## Abstract

**Background:**

Recent discoveries on Palau are claimed to represent the remains of small-bodied humans that may display evidence insular size reduction. This claim has yet to be statistically validated

**Methodology/Principal Findings:**

Published postcranial specimens (*n* = 16) from Palau were assessed relative to recent small-bodied comparative samples. Resampling statistical approaches were employed to test specific hypotheses relating to body size in the Palau sample. Results confirm that the Palau postcranial sample is indisputably small-bodied.

**Conclusions/Significance:**

A single, homogenous body size morph is represented in early prehistoric postcrania from Palau. Small body size in early Palauans is an ancestral characteristic and was likely not a consequence of in-situ size reduction. Specimens from Palau have little bearing upon hypothesised insular size reduction in the ancestral lineage of *Homo floresiensis*.

## Introduction

Body size variation in Southeast Asian terminal Pleistocene and Holocene humans is poorly understood. Present inhabitants of the SE Asian tropics display considerable body size variability [1]–[3]. ‘Negritos’ of the Philippines and surrounding Islands are distantly related to Polynesian peoples and approach African Pygmies in their diminutive size [1], [4]. Archaeological material from Palau is pertinent to establishing a timeframe for the dispersal of small-bodied humans in Southeast Asia [5]–[7]. In a recent contribution, Berger et al. [8] concluded that specimens from archaeological sites in the Rock Islands display evidence of chronological size reduction. This was argued to be a function of significant ecological selection in a reproductively-isolated population [8].

Berger et al. [8] propose that postcranial elements recovered from Ucheliungs and Omodokel caves (*n* = 61) sample the lowest extremes of extant size variation in *Homo sapiens* (Table 1). This proposal has been the focus of contention within scientific circles [9], [10]. Statistical appraisal of size variability within the skeletal sample from Palau [7], [8] is imperative to understanding patterns and homogeneity of body size distribution in early prehistoric inhabitants of Micronesia and in validating any hypothesis of insular dwarfism [8].

**Table 1:** Skeletal inventory from Palau used in the analysis.

Specimen	Locality	BIEPIC	HDAB	ACET	FHD	PTB
B:OR-14:8-991	Ucheliungs		34.90			
B:OR-15:18-014	Omedokel		32.90			
B:OR-15:18-015	Omedokel		34.90			
B:OR-15:18-024	Omedokel		44.10			
B:OR-15:18-054	Omedokel		41.20			
B:OR-15:18-088	Omedokel		42.40			
B:OR-15:18-009	Omedokel			39.50		
B:OR-15:18-087	Omedokel			46.10		
B:OR-15:18-013	Omedokel				36.10	
B:OR-15:18-098	Omedokel				38.80	
B:OR-14:8-003	Ucheliungs					63.10
B:OR-15:8-040	Omedokel					53.10
TU-1-L8	Chelechol ra Orrak	57.80				
TU-1-L10r	Chelechol ra Orrak	58.50				
TU-1-1	Chelechol ra Orrak				36.10	
TU-1-L9-1	Chelechol ra Orrak					64.80
Liang Bua 1	Liang Bua			36.00	31.50	51.50

Humeral bi-epicondylar breadth [BIEPIC]; Humeral distal articular breadth [HDAB]; Acetabulum diameter [ACET]; Femoral head diameter [FHD]; Proximal tibia articular breadth [PTB].

This correspondence focuses upon two explicit hypotheses concerning body size in prehistoric humans from Palau. Does the skeletal sample from Palau [7], [8] fall within observed morphological size ranges of extant small-bodied humans and is the skeletal sample homogenous or heterogenous? An explicit assessment of an associated femur and tibia from Chelechol ra Orrak [7] is pertinent. Simply stated, is there overwhelming evidence for a single, small-bodied morphotype among the earliest inhabitants of Palau?

## Results

**Figure 1:**
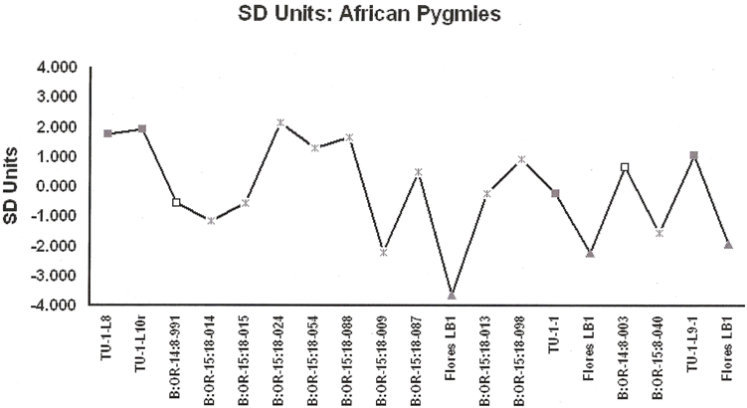
Specimen inventory numbers (see Table 1 for inventory) are from Nelson and Fitzgerald (2006; TU [Chelechol ra Orrak]), Berger et al. (2008; B:OR-14 [Ucheliungs], B:OR-15 [Omedokel]) and Brown et al. (2004; LB1). Chelechol ra Orrak, Grey Squares; Ucheliungs Cave, White Squares; Omedokel Cave, Grey Asterisks; Liang Bua 1, Grey Triangles.

**Figure 2:**
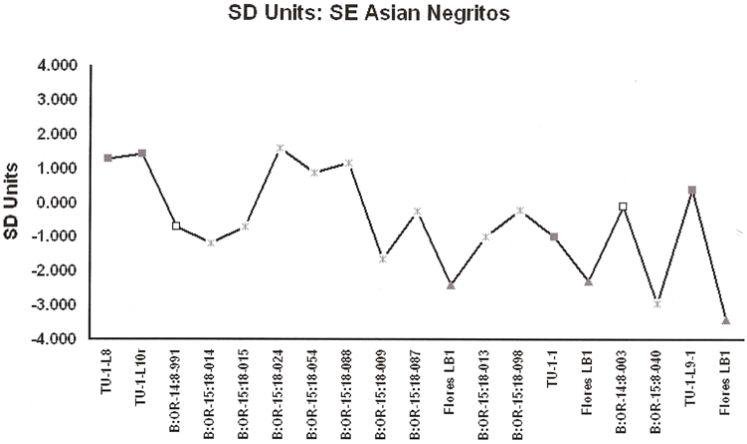
Specimen inventory numbers (see Table 1 for inventory) are from Nelson and Fitzgerald (2006; TU [Chelechol ra Orrak]), Berger et al. (2008; B:OR-14 [Ucheliungs], B:OR-15 [Omedokel]) and Brown et al. (2004; LB1). Chelechol ra Orrak, Grey Squares; Ucheliungs Cave, White Squares; Omedokel Cave, Grey Asterisks; Liang Bua 1, Grey Triangles.

Results are consistent irrespective of whether bootstrapping or randomization is preferred [Supplementary Data 1]. African Pygmies and Southeast Asian Negritos are remarkably similar in their distal humerii but differ significantly in lower limb size profiles (Figures 1 and 2 [Tables S1 and S2]). The African Pygmy and Southeast Asian Negrito samples are significantly smaller than African and European samples [Tables S3 and S4]. Direct comparisons confirm that a majority of the Palau postcrania derive from individuals whom can be accommodated within the observed size ranges of small-bodied humans (Tables 2 and 3). Four distal humerii exceed the upper 95% CI's for African Pygmies, but not SE Asian Negritos. Lower limb specimens are not excessively small and approximate the observed distribution of African Pygmies. Two lower limb elements; B:OR-15:18-009 [innominate] and B:OR-15:18-040 [proximal tibia] are ‘extremely small’. While the B:OR-15:18-040 proximal tibia approximates the LB1 hominin [11] there is little support for the hypothesis that these specimens exceed the lower size range in recent humans. In contrast, postcranial dimensions of the LB1 hominin cannot be sampled from recent small-bodied humans and are truly diminutive (Tables 2 and 3). Results confirm previous conclusions that the Rock Island specimens are those of small-bodied humans [8].

**Table 2:** Pairwise randomization comparisons of Palau and LB1 specimens.

Comparisons	Parameter	Sample A	Sample B	Obs Diff	Rand Diff	5% CI	95% CI	P-value	*Student Conf*
African Pygmy V's TU-1-L8	BIEPIC	50.54	57.80	7.261	3.641	0.508	6.932	**0.025**	Not Significant
African Pygmy V's TU-1-L10r	BIEPIC	50.54	58.50	7.961	3.754	0.532	6.956	**0.026**	Not Significant
African Pygmy V's B:OR-14:8-991	HAB	36.83	34.90	1.925	2.861	0.195	6.094	0.670	Confirm
African Pygmy V's B:OR-15:18-014	HAB	36.83	32.90	3.925	2.908	0.264	6.025	0.289	Confirm
African Pygmy V'sB:OR-15:18-015	HAB	36.83	34.90	1.925	2.875	0.195	6.094	0.672	Confirm
African Pygmy V's B:OR-15:18-024	HAB	36.83	44.10	7.275	2.999	0.122	6.551	**0.001**	Confirm
African Pygmy V's B:OR-15:18-054	HAB	36.83	41.20	4.375	2.886	0.053	2.611	0.264	Confirm
African Pygmy V's B:OR-15:18-088	HAB	36.83	42.40	5.575	2.973	0.095	6.392	**0.010**	Not Significant
African Pygmy V's B:OR-15:18-009	ACET	44.49	39.50	4.986	2.092	0.026	5.368	0.079	Confirm
African Pygmy V's B:OR-15:18-087	ACET	44.49	46.10	1.614	2.020	0.085	5.148	0.576	Confirm
African Pygmy V's Flores LB1	ACET	44.49	36.00	8.486	2.227	0.132	5.836	**0.016**	Confirm
African Pygmy V's B:OR-15:18-013	FHD	36.65	36.10	0.547	1.936	0.104	4.913	0.817	Confirm
African Pygmy V's B:OR-15:18-098	FHD	36.65	38.80	2.152	1.965	0.166	4.831	0.518	Confirm
African Pygmy V's TU-1-1	FHD	36.65	36.10	0.547	1.953	0.104	4.913	0.814	Confirm
African Pygmy V's Flores LB1	FHD	36.65	31.50	5.147	2.034	0.035	5.147	**0.042**	Confirm
African Pygmy V's B:OR-14:8-003	PTB	60.09	63.10	3.012	3.693	0.523	9.665	0.568	Confirm
African Pygmy V's B:OR-15:8-040	PTB	60.09	53.10	6.988	3.864	0.323	9.265	0.123	Confirm
African Pygmy V's TU-1-L9-1	PTB	60.09	64.80	4.712	3.771	0.591	9.733	0.259	Confirm
African Pygmy V's Flores LB1	PTB	60.09	51.50	8.588	3.943	0.387	9.201	0.087	Confirm

**Table 3:** Pairwise randomization comparisons of Palau and LB1 specimens.

Comparisons	Parameter	Sample A	Sample B	Obs Diff	Rand Diff	5% CI	95% CI	P-value	*Student Conf*
SE Asian Negrito V's TU-1-L8	BIEPIC	51.53	57.80	6.270	4.098	0.616	9.314	0.249	Confirm
SE Asian Negrito V's TU-1-L10r	BIEPIC	51.53	58.50	6.970	4.138	0.698	9.330	0.174	Confirm
SE Asian Negrito V's B:OR-14:8-991	HAB	37.93	34.90	3.034	3.271	0.568	6.994	0.461	Confirm
SE Asian Negrito V's B:OR-15:18-014	HAB	37.93	32.90	5.034	3.239	0.767	6.948	0.217	Confirm
SE Asian Negrito V's B:OR-15:18-015	HAB	37.93	34.90	3.034	3.227	0.782	6.994	0.453	Confirm
SE Asian Negrito V's B:OR-15:18-024	HAB	37.93	44.10	6.166	3.293	0.569	7.208	0.127	Confirm
SE Asian Negrito V's B:OR-15:18-054	HAB	37.93	41.20	3.266	3.256	0.636	7.396	0.399	Confirm
SE Asian Negrito V's B:OR-15:18-088	HAB	37.93	42.40	4.466	3.262	0.608	7.169	0.263	Confirm
SE Asian Negrito V's B:OR-15:18-009	ACET	48.73	39.50	9.225	3.986	0.617	8.269	**0.010**	Not Significant
SE Asian Negrito V's B:OR-15:18-087	ACET	48.73	46.10	2.265	3.693	0.434	8.049	0.585	Confirm
SE Asian Negrito V's Flores LB1	ACET	48.73	36.00	12.725	4.067	0.501	8.386	**0.010**	Confirm
SE Asian Negrito V's B:OR-15:18-013	FHD	39.54	36.10	3.439	3.006	0.280	6.231	0.416	Confirm
SE Asian Negrito V's B:OR-15:18-098	FHD	39.54	38.80	0.739	2.697	0.263	6.171	0.852	Confirm
SE Asian Negrito V's TU-1-1	FHD	39.54	36.10	3.439	3.024	0.280	6.231	0.416	Confirm
SE Asian Negrito V's Flores LB1	FHD	39.54	31.50	8.039	3.191	0.292	7.691	**0.021**	Confirm
SE Asian Negrito V's B:OR-14:8-003	PTB	63.47	63.10	0.370	4.665	0.247	11.513	0.908	Confirm
SE Asian Negrito V's B:OR-15:8-040	PTB	63.47	53.10	10.370	4.961	0.475	11.243	0.061	Confirm
SE Asian Negrito V's TU-1-L9-1	PTB	63.47	64.80	1.330	4.691	0.293	11.560	0.822	Confirm
SE Asian Negrito V's Flores LB1	PTB	63.47	51.50	11.970	5.045	0.354	11.970	**0.045**	Confirm

**Figure 3:**
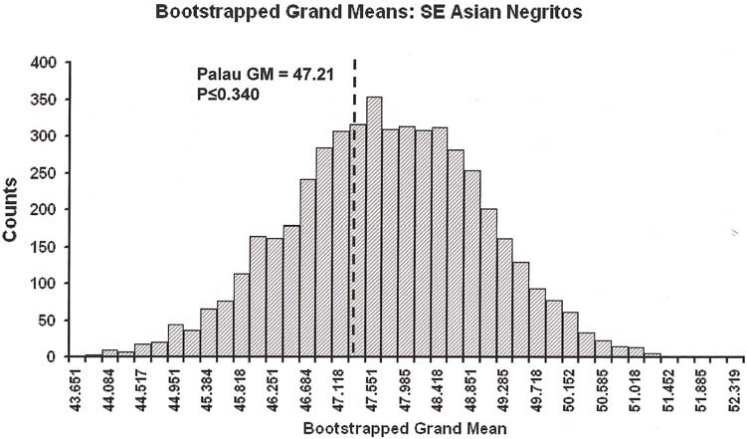
Bootstrapped Grand Means (n = 5000) for the SE Asian Negritos.

The CV* for the distal humerii approaches the significance criterion of African Pygmy and SE Asian Negrito CV*s but remains insignificant (Table 4 [Figures S1 & S2]). The Grand Mean of the Palau series (*n* = 16) is significantly greater than approximates derived from African Pygmies but not Southeast Asian Negritos (Table 4; Figures 3 and S3). Bootstrapped standard deviations (SD's) confirm that the variation within the Palau postcranial series is not significant and is generally consistent with that observed in small-bodied human references (Table 4). Statistical comparisons of the associated femur and tibia from Chelechol ra Orrak [7] confirms that these derive from a small-bodied individual [Figures S4 and S5]. Pairwise randomization and bootstrapped t-tests reject the hypothesis that the Chelechol ra Orrak specimens exceed the size range of small-bodied humans. The observed homogeneity of prehistoric humans from Palau confirms the hypothesis that a single size morph is represented at c3000 BP. Early prehistoric Palauans were indisputably ‘small-bodied’.

**Table 4:** Resampling models for Palau specimens.

*Palau V's African Pygmies*							
Comparison	CV*	Grand Means	St Dev (Geo Means)	OMD Femur Lth	Student's t (Femur Lth)	OMD Tibia Lth	Student's t (Tibia Lth)
Palau	12.786	47.21	11.10	392.00		318.00	
African Pygmies	9.208	45.45	9.91	370.82		318.60	
Obs Diff/Students t		1.760		21.180	0.954	0.600	−0.027
<Obs Palau	4658	4860	4287	3410	2353	2033	2435
Significance	0.9316	**0.9720**	0.8574	0.6821	0.4707	0.4067	0.4871

*Palau V's SE Asian Negritos*							
Comparison	CV*	Grand Means	St Dev (Geo Means)	OMD Femur Lth	Student's t (Femur Lth)	OMD Tibia Lth	Student's t (Tibia Lth)
Palau	12.786	47.21	11.10	392.00		318.00	
SE Asian Negritos	10.746	46.76	10.34	396.91		337.10	
Obs Diff/Students t		0.450		4.991	−0.199	19.105	−0.865
<Obs Palau	3785	1701	3272	837	2588	2933	2647
Significance	0.7570	0.3403	0.6545	0.1676	0.5177	0.5867	0.5295

## Discussion

Ongoing investigations confirm an initial human presence on the southern Islands of the Palau archipelago by c3000 BP [5]–[8]. Berger et al. [8] proposed that the earliest inhabitants of the Rock Islands were small-bodied and this is confirmed by statistical analyses of available postcranial specimens. Two lower limb elements are unarguably ‘very small’ but even these small individuals do not approximate the diminutive size of the LB1 *H. floresiensis* holotype [11], [12].

Analyses of within-group variability confirm that Palauan postcrania are neither excessively variable nor excessively small. All specimens included in this study approximate the size ranges of African Pygmies and Southeast Asian Negritos [13]. Statistical tests further confirm the remarkable homogeneity of size in prehistoric Palauans. Upper and lower limb elements from Chelechol ra Orrak are not consistently larger than corresponding specimens from the Rock Island sites [7], [8]. An associated femur and tibia from Chelechol ra Orrak are within the size range of small-bodied humans. Results of this study provide no support for the hypothesis that two distinct size ‘morphs’ are represented at c3000 BP. Evidence overwhelmingly supports the conclusion that the pioneer colonists of Palau were small-bodied.

Berger et al. [8] hypothesized body size reduction among the Rock Island samples. Current contextual and chronological ambiguity at Omedokel cave compromises a hypothesis of insular dwarfism [8]. Published AMS dates are wildly disparate and contrast with the chronological controls evident at Ucheliungs [8]. Results overwhelmingly support the proposal that the earliest colonists of Palau were small-bodied and that within-situ size reduction cannot be substantiated by current evidence. Body size reduction in the ancestral population of earliest colonists of Palau is likely unconnected to the prolonged genetic isolation envisaged for the Middle Pleistocene of Flores [11], [12], [14]–[16].

## Materials and Methods

Distal humeral, innominate, proximal femoral and proximal tibial specimens from archaeological sites dating to c3000 BP and younger (Table 1; 7, 8) and corresponding dimensions from Liang Bua 1 [11] were compared with two small-bodied reference samples. Resampling approaches were utilised [17], [18]. Articular dimensions were the focus of this analysis [19]–[22]. African Pygmy (*n* = 34) and SE Asian Negrito (*n* = 44) specimens are derived from several Institutions [Supplementary Data 1]. Resampling comparisons were performed with and without replacement [17], [18]. Initially, absolute deviations of individual specimens (table 1) from the arithmetic mean of the reference samples were randomization over *n* = 5000 iterations. These comparisons were performed using Rundom Projects 2 [23]; http://pjadw.tripod.com]. Computational analyses of bootstrapped Student's t' in the case of a single observation [24: 227–228] was computed using Resampling Stats for Excel 2003 [25].

The CV*, modified for small sample sizes [24], for available distal humerii (*n* = 6) can facilitate direct assessment within a single dimension. A bootstrapping approach repeatedly selected six specimens from the comparative series to calculate the CV* over 5000 iterations [26], [27]. The test statistic is a ratio specifying the position of the observed CV* within a generated distribution of CV*s of the comparative distributions (*n* = 5000). A modified version of the approach outlined in Green et al. [28] was used to assess variation within the postcranial series (*n* = 16). Geometric Means were calculated [24] for and the Grand Mean and standard deviation (SD) were used as proxies of within-sample variance. Grand Means and SD's were bootstrapped 5000 times. Femoral and tibial lengths of the associated skeleton from Chelechol ra Orrak [7] were subjected to the test procedures as outlined previously.

## Supporting Information

Table S1 Pooled-Sex descriptive statistics for the African Pygmy and Southeast Asian Negrito samples
(0.04 MB RTF)

Table S2 Randomization comparisons of the small-bodied comparatives.
(0.03 MB RTF)

Table S3 Randomization comparisons of the small-bodied comparatives.
(0.04 MB RTF)

Table S4 Randomization comparisons of the small-bodied comparatives.
(0.04 MB RTF)

Figure S1 (3.68 MB TIF)

Figure S2 (4.13 MB TIF)

Figure S3 (4.10 MB TIF)

Figure S4 (3.35 MB TIF)

Figure S5 (3.88 MB TIF)
