# Supplementary material for: Size Variation in Small-Bodied Humans from Palau, Micronesia
Source: PLoS One. 2008 Dec 17;3(12):e3939. doi: 10.1371/journal.pone.0003939 (PMC2596964; doi:10.1371/journal.pone.0003939)
Supplement: Table S1 — Pooled-Sex descriptive statistics for the African Pygmy and Southeast Asian Negrito samples (0.04 MB RTF) [file pone.0003939.s001.rtf]

Supplementary Table 1. Pooled-Sex descriptive statistics for the African  Pygmy and Southeast Asian Negrito samples

						
African Pygmies						
	BIEPIC	DAW	ACET	FHD	PTB	
Mean	50.54	36.83	43.75	36.65	60.09	
SD	4.13	3.39	2.05	2.34	4.46	
Maximum	57.31	43.40	47.99	41.62	67.34	
Minimum	44.08	30.87	39.34	33.09	49.96	
CV (%)	8.18	9.21	4.69	6.39	7.43	
						
Southeast Asian Negritos						
	BIEPIC	DAW	ACET	FHD	PTB	
Mean	51.53	37.70	47.18	39.54	63.47	
SD	4.90	4.05	4.66	3.52	5.59	
Maximum	61.89	45.56	56.24	45.56	71.71	
Minimum	41.58	28.21	38.87	31.64	51.02	
CV (%)	9.50	10.75	9.88	8.90	8.81	

Humeral bi-epicondylar breadth [BIEPIC]; Humeral distal articular breadth [HDAB]; Acetabulum diameter [ACET]; Femoral head diameter [FHD]; Proximal tibia articular breadth [PTB]
