# Supplementary material for: Size Variation in Small-Bodied Humans from Palau, Micronesia
Source: PLoS One. 2008 Dec 17;3(12):e3939. doi: 10.1371/journal.pone.0003939 (PMC2596964; doi:10.1371/journal.pone.0003939)
Supplement: Table S2 — Randomization comparisons of the small-bodied comparatives. (0.03 MB RTF) [file pone.0003939.s002.rtf]

Supplementary Table 2: Randomization comparisons of the small-bodied comparatives

Comparisons	Parameter	Sample A	Sample B	Obs Diff	Rand Diff	5% CI	95% CI	P-value	
African Pygmy V's SE Asian Negritos	BIEPIC	50.54	51.53	0.991	0.887	0.068	2.178	0.375	
African Pygmy V's SE Asian Negritos	HAB	36.83	37.70	1.109	0.691	0.048	1.685	0.203	
African Pygmy V's SE Asian Negritos	ACET	43.75	47.18	4.239	0.843	0.073	2.073	0.001	
African Pygmy V's SE Asian Negritos	FHD	36.65	39.54	2.891	0.626	0.052	1.519	0.001	
African Pygmy V's SE Asian Negritos	PTB	60.09	63.47	3.382	1.130	0.102	0.271	0.013	
