# Supplementary material for: Size Variation in Small-Bodied Humans from Palau, Micronesia
Source: PLoS One. 2008 Dec 17;3(12):e3939. doi: 10.1371/journal.pone.0003939 (PMC2596964; doi:10.1371/journal.pone.0003939)
Supplement: Table S3 — Randomization comparisons of the small-bodied comparatives. (0.04 MB RTF) [file pone.0003939.s003.rtf]

Supplementary Table 3: Randomization comparisons of the small-bodied comparatives

Comparisons	Parameter	Sample A	Sample B	Obs Diff	Rand Diff	5% CI	95% CI	P-value	
African Pygmy V's African Bantu	BIEPIC	50.54	58.65	8.108	0.836	0.063	2.091	0.001	
African Pygmy V's African Bantu	HAB	36.83	42.74	5.911	0.677	0.050	1.649	0.001	
African Pygmy V's African Bantu	ACET	43.75	51.74	7.995	0.772	0.063	1.842	0.001	
African Pygmy V's African Bantu	FHD	36.65	42.98	6.333	0.702	0.057	1.710	0.001	
African Pygmy V's African Bantu	PTB	60.09	70.66	10.572	1.109	0.087	2.665	0.001	
African Pygmy V's Medieval Hungarians	BIEPIC	50.54	59.29	8.749	1.072	0.088	2.639	0.001	
African Pygmy V's Medieval Hungarians	HAB	36.83	43.19	6.360	0.807	0.065	1.947	0.001	
African Pygmy V's Medieval Hungarians	ACET	43.75	52.65	8.907	0.984	0.082	2.420	0.001	
African Pygmy V's Medieval Hungarians	FHD	36.65	44.24	7.594	0.838	0.071	2.013	0.001	
African Pygmy V's Medieval Hungarians	PTB	60.09	69.68	9.595	1.183	0.094	2.944	0.001	
