# Supplementary material for: Size Variation in Small-Bodied Humans from Palau, Micronesia
Source: PLoS One. 2008 Dec 17;3(12):e3939. doi: 10.1371/journal.pone.0003939 (PMC2596964; doi:10.1371/journal.pone.0003939)
Supplement: Table S4 — Randomization comparisons of the small-bodied comparatives. (0.04 MB RTF) [file pone.0003939.s004.rtf]

Supplementary Table 4: Randomization comparisons of the small-bodied comparatives

Comparisons	Parameter	Sample A	Sample B	Obs Diff	Rand Diff	5% CI	95% CI	P-value	
SE Asian Negritos V's African Bantu	BIEPIC	51.53	58.65	7.118	0.755	0.054	1.856	0.001	
SE Asian Negritos V's African Bantu	HAB	37.70	42.74	5.035	0.590	0.048	1.448	0.001	
SE Asian Negritos V's African Bantu	ACET	47.18	51.74	4.506	0.706	0.054	1.756	0.001	
SE Asian Negritos V's African Bantu	FHD	39.54	42.98	3.441	0.560	0.047	1.379	0.001	
SE Asian Negritos V's African Bantu	PTB	63.47	70.66	7.190	0.886	0.073	2.148	0.001	
SE Asian Negritos V's Medieval Hungarians	BIEPIC	51.53	59.29	7.759	0.950	0.070	2.315	0.001	
SE Asian Negritos V's Medieval Hungarians	HAB	37.70	43.19	5.484	0.701	0.060	1.681	0.001	
SE Asian Negritos V's Medieval Hungarians	ACET	47.18	52.65	5.472	0.906	0.067	2.240	0.001	
SE Asian Negritos V's Medieval Hungarians	FHD	39.54	44.24	4.703	0.668	0.051	1.641	0.001	
SE Asian Negritos V's Medieval Hungarians	PTB	63.47	69.68	6.212	0.985	0.079	2.415	0.001	
