# Supplementary figures and images for: Size Variation in Small-Bodied Humans from Palau, Micronesia
Source: PLoS One. 2008 Dec 17;3(12):e3939. doi: 10.1371/journal.pone.0003939 (PMC2596964; doi:10.1371/journal.pone.0003939)

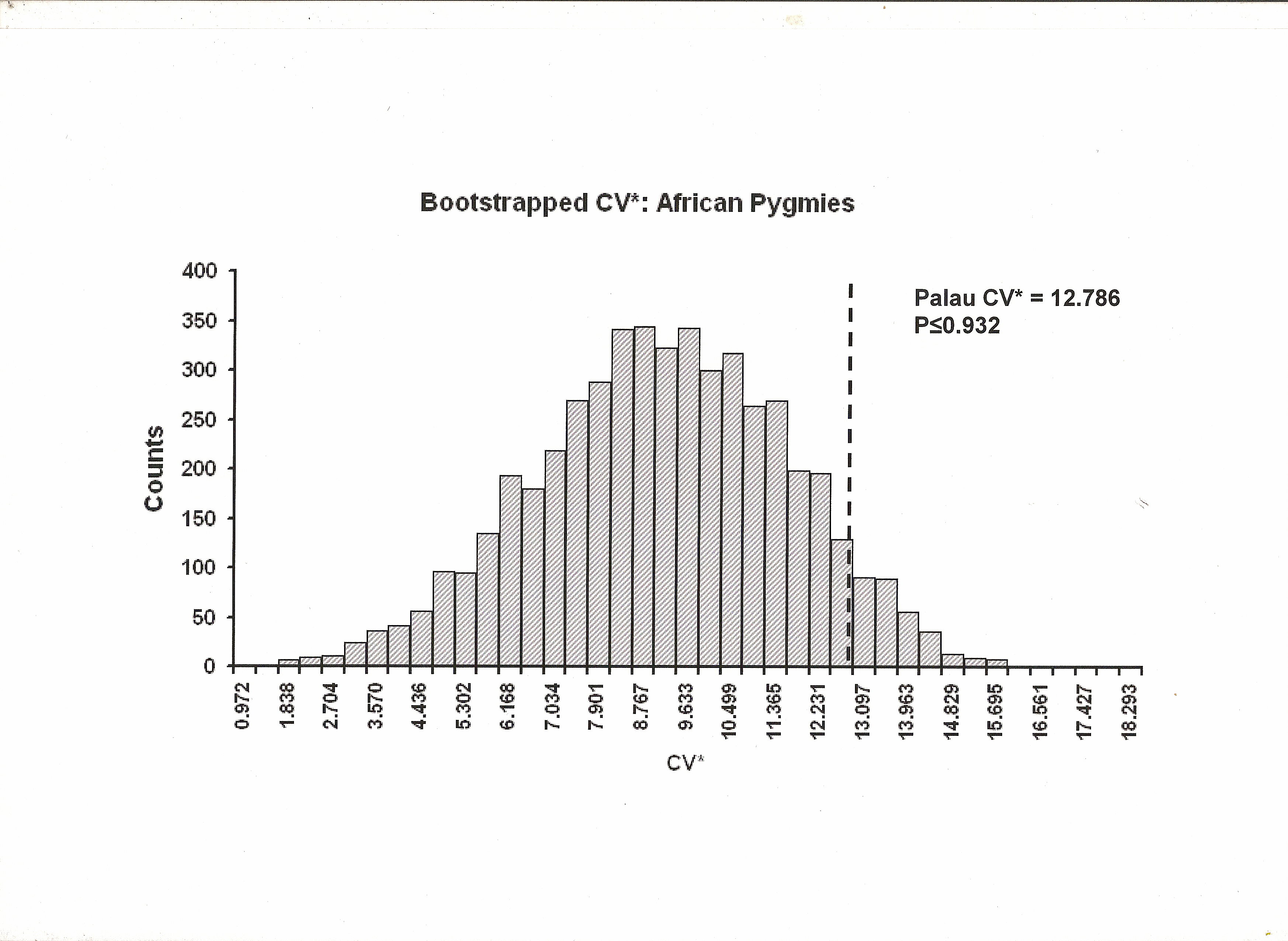

Supplement: Figure S1 — (3.68 MB TIF) [file pone.0003939.s005.tif]

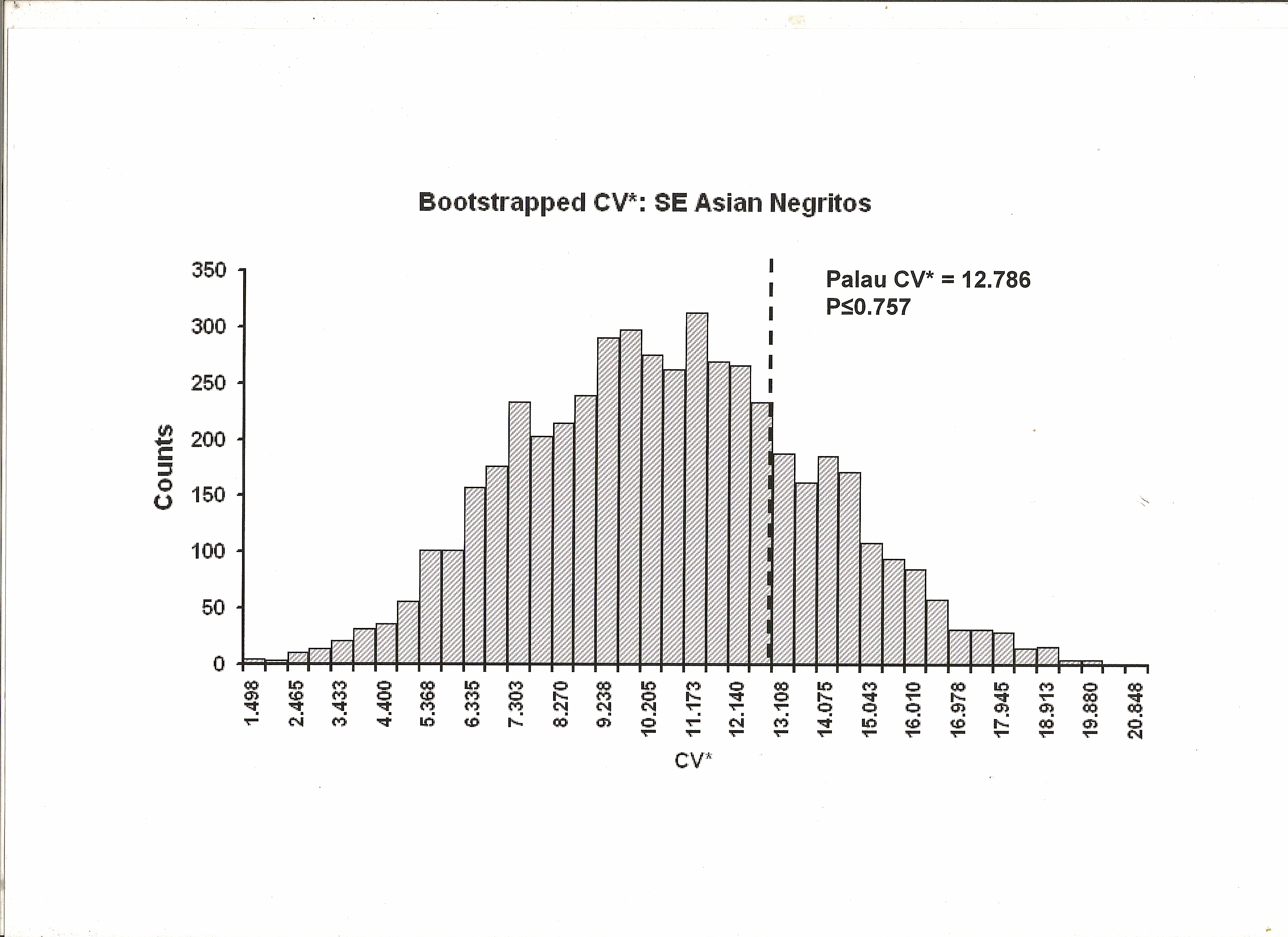

Supplement: Figure S2 — (4.13 MB TIF) [file pone.0003939.s006.tif]

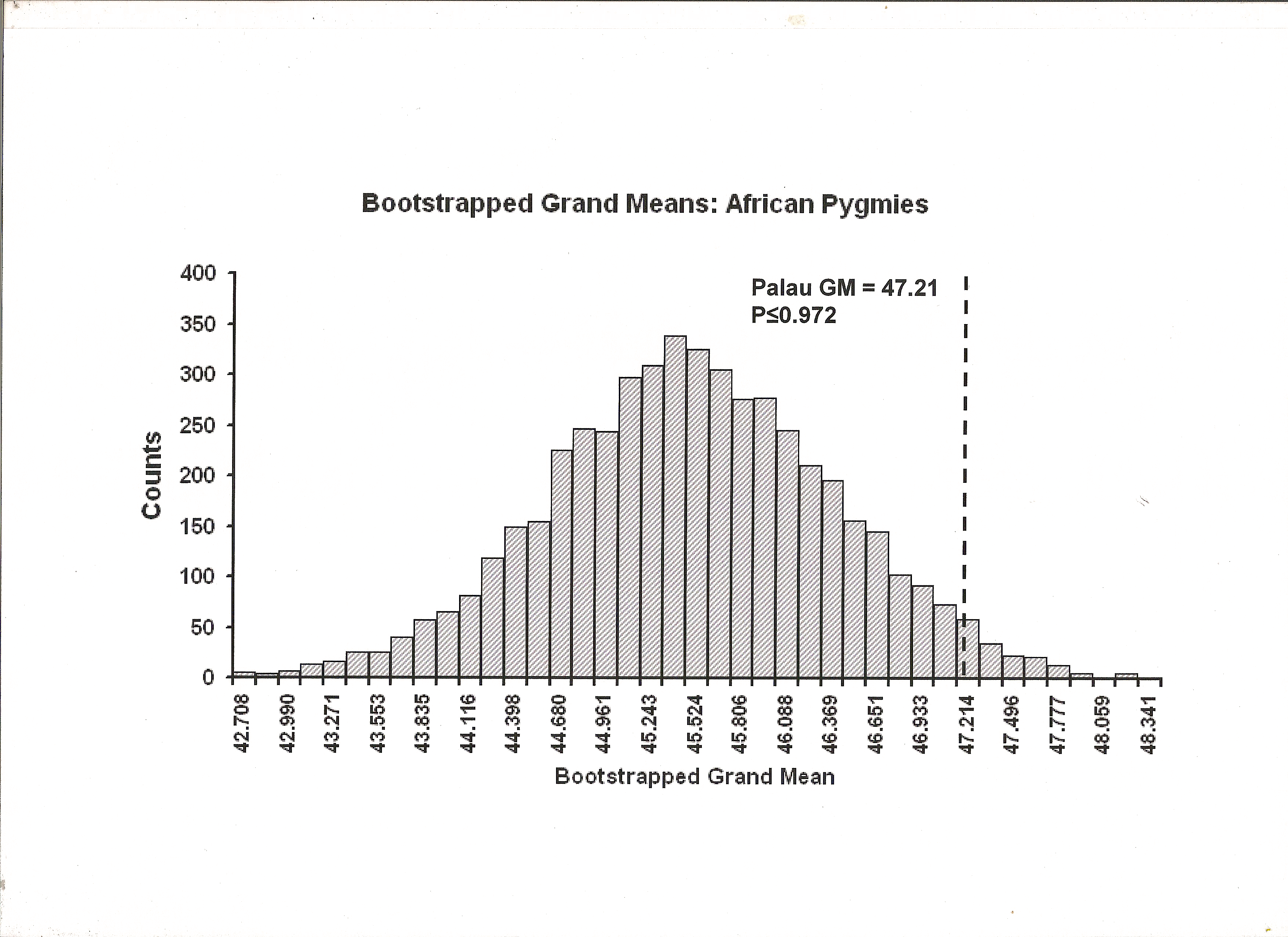

Supplement: Figure S3 — (4.10 MB TIF) [file pone.0003939.s007.tif]

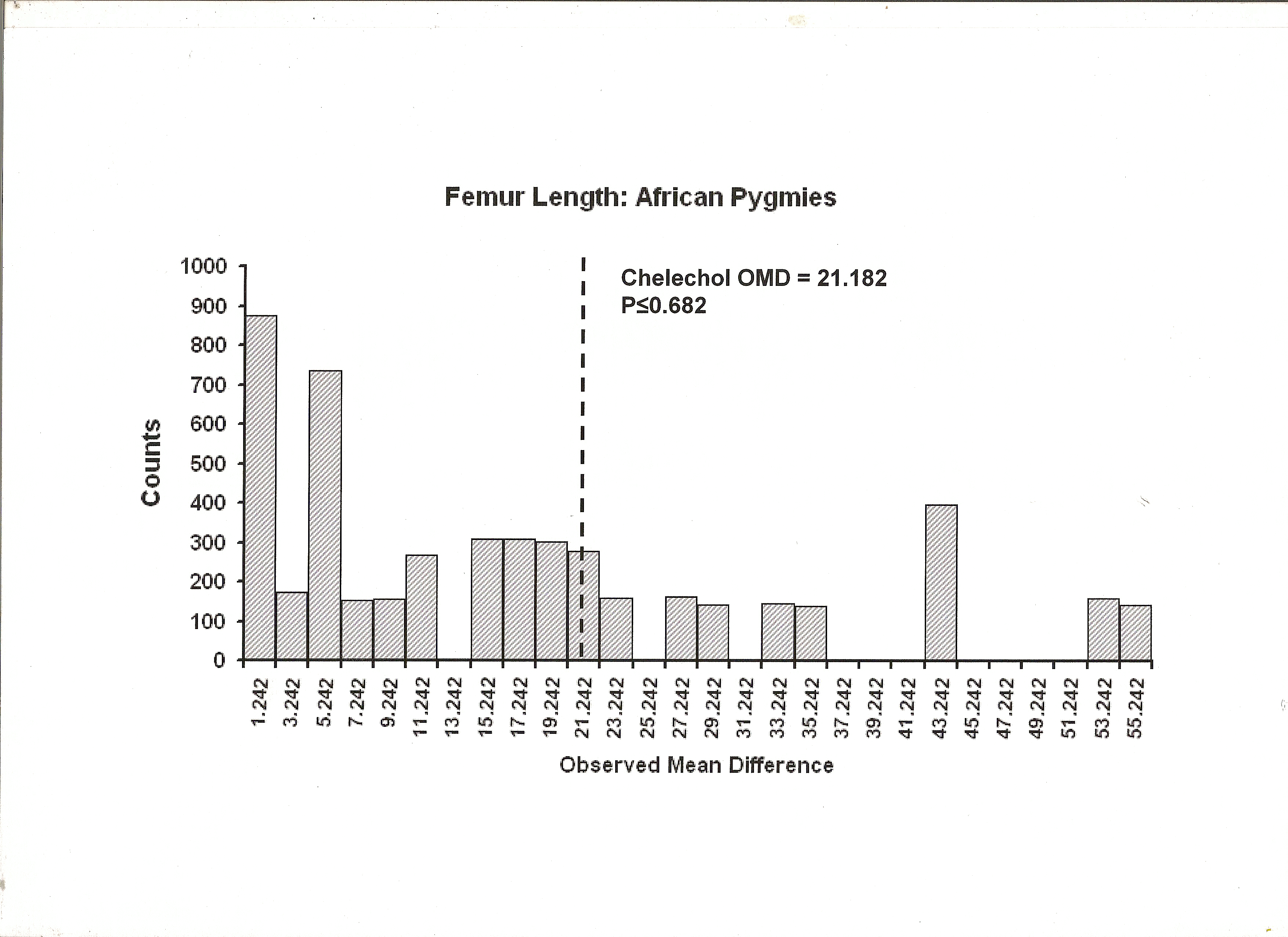

Supplement: Figure S4 — (3.35 MB TIF) [file pone.0003939.s008.tif]

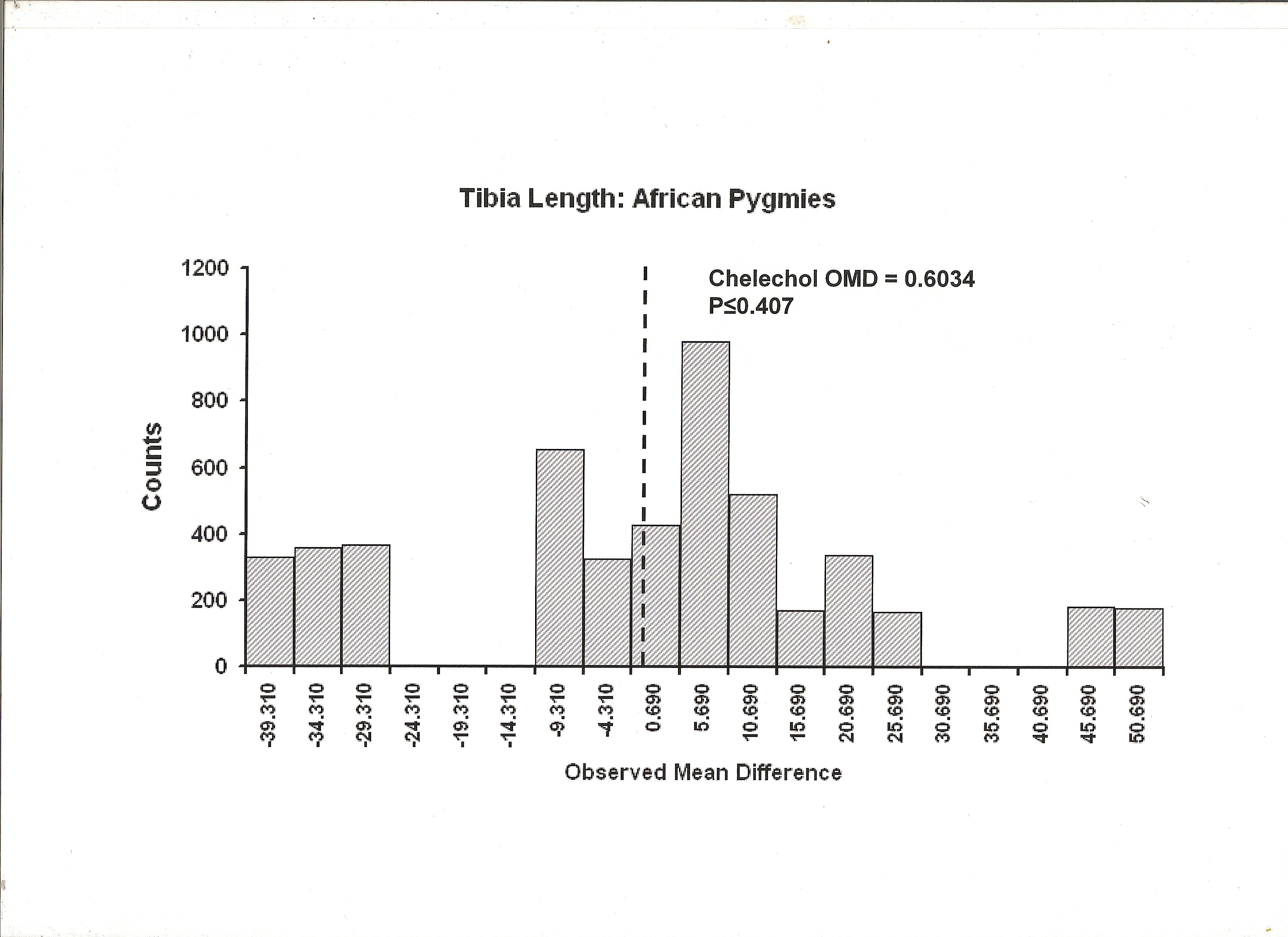

Supplement: Figure S5 — (3.88 MB TIF) [file pone.0003939.s009.tif]
